# Patient satisfaction with pharmacy services among users and non users of community based health insurance scheme at public health facilities in Gamo Zone, South Ethiopia: a comparative cross sectional study

**DOI:** 10.1186/s40780-024-00350-0

**Published:** 2024-06-07

**Authors:** Fitsum Teferi Gulta, Tidenek Mulugeta, Biruk Wogayehu, Mende Mensa

**Affiliations:** 1 Department of Pharmacy, Arbaminch College Health Sciences, Arba Minch, Ethiopia; 2 Department of Pharmaceutical Supply Chain Management, School of Pharmacy, Jimma University, Oromia Region, Jimma, Ethiopia; 3 Department of pharmacy, Arbaminch University, Arba Minch, Ethiopia

**Keywords:** Community based health insurance, Patient satisfaction, Pharmacy services, Ethiopia

## Abstract

**Background:**

Patient satisfaction is a crucial humanistic outcome metric in pharmacy services. There was a lack of evidence on patients’ satisfaction with pharmacy services in Gamo zone among users and nonusers of the CBHI scheme. Therefore, the aim this study is to compare the level of patient satisfaction with pharmacy services among users and nonusers of community based health insurance scheme at public health facilities in Gamo zone, South Ethiopia.

**Method:**

A facility based comparative cross sectional study design with mixed approach was conducted from June 1 to 30, 2023. A total of 522 study participants and 16 key informants were included as the sample size for quantitative and qualitative study, respectively. The quantitative data was gathered from the study participants who visited the outpatient pharmacy department during the study period by using a simple random sampling technique, while the purposive sampling technique was used to select clients and key informants for the qualitative study. The adjusted odds ratio (AOR) was used to measure the association between independent variables and patient satisfaction toward outpatient pharmacy services at the P values < 0.05 and 95% confidence interval (CI).

**Results:**

From the total of study participants, 195 (73.9%) of insured and 175 (67.8%) of noninsured clients were satisfied with pharmacy services offered at public health facilities. The gender of insured (95% CI = 2.00-12.36, (p 0.01)), and noninsured (95% CI = 0.658–2.881, (p 0.02)), waiting time of insured (95% CI = 0.057–0.766, (p 0.0027)), and noninsured (95% CI = 0.084–0.925, (p 0. 0021)) and premium affordability of insured (95% CI = 0.0605-4.860, (p 0.00)) were significantly associated factors with client satisfaction at *p* < 0.05 and 95% CI. Based on qualitative finding, as member of the CBHI scheme, the members had a greater opportunity to receive a good pharmacy services, because they were more familiar with the physicians and the institutions.

**Conclusion:**

The clients with insurance perceived high level of satisfaction with pharmacy services in public health facilities than noninsured. The gender and waiting times at outpatient pharmacy department for both groups of study participants and the premium affordability for the insured groups of clients were factors affecting client satisfactions with pharmacy services.

## Background

The Community Based Health Insurance (CBHI) program is a non-profit form of health insurance that has been utilized by the underprivileged to safeguard themselves against the high costs of obtaining health services and treatment for illness [1]. Health insurance is a way to protect people from catastrophic financial losses in the case of a serious illness by distributing the risk of having to pay for medical expenses among a group of people or households [2]. The community based health insurance scheme promise better access to healthcare and risk protection for low-income households against the cost of illness [3].

The global community has advocated for a CBHI scheme to improve access to healthcare services and thus achieve universal health coverage (UHC) over the last few decades [3]. The poor have used the community-based health insurance program to shield themselves from the high expenses of disease treatment and medical care [4].

The Ethiopian government formulated and implemented a health finance plan in 1998, which allocates funds for the health sector to be raised from different sources and allows the government to provide health services through its health facilities through a consumer-shared cost structure[5]. The CBHI scheme was first implemented in Ethiopia in June 2011 as a pilot in 13 districts spread across four regions: Tigray, Amhara, Oromia, and the South Nations and Nationalities People’s Region (SNNPR). The systems implemented in 13 areas have yielded preliminary findings that show promise. Although the coverage differed throughout the pilot regions, the average CBHI enrollment percentage was above 50% overall. 74.6% coverage rate was achieved in the Southern Ethiopian area [6]. Thirty two million people, or about seven million households, signed up for the CBHI program between 2015 and 2020 [7].

The premium payment for the scheme in Ethiopia varies by region, ranging from 10.50 to 15.50 Ethiopian birr per month per household. CBHI officials and community leaders at the Kebele level have the authority to change premium collecting intervals based on local conditions [6]. The scheme is enrolled at the household level rather than on an individual basis [8, 9]. The federal government pays a 25% general subsidy to all members, while regional governments share the costs of granting a fee waiver to the poorest 10% of the population [6, 9]. As of 2020, 245 hospitals and 1920 healthcenters nationwide are contractually providing health services to CBHI users [7].

The CBHI’s Ethiopian benefit packages offer all family health treatments and curative care that are part of the essential health package, with the exception of dental implantation and optical services [6]. The health insurance plan was created as a way to improve pharmacy services including the affordability of medicines and other supplies for its members while also increasing their appropriate use [10]. Pharmacy service is provided by pharmacists at the health facilities on prescription medication dispensing, non-prescription medication dispensing, dispensing sanitary items, patient counseling, and patient advice during discharge [11]. The availability of medications and physicians, and the quality of pharmacy services received by enrollees are all positively correlated with CBHI satisfaction [12, 13].

A survey conducted in Nigeria on Nigerian national health insurance scheme showed that, over half of patients assessed their pharmacy services offering as unsatisfactory in comparison to their ideal of the amount of time pharmacists provided to spend with them [14]. Another study done at Boru Meda Hospital, Northeast Ethiopia revealed that the cleanliness of the pharmacy received the highest satisfaction rating from CBHI member clients during pharmacy service delivery, while the availability of drugs received the lowest satisfaction rating. Additionally, the long waiting time for a physicians and pharmacist’s consultation has a negative effect on patient satisfaction [15].

As far as the researcher knowledge, there is a limited research done to assess the factors and reasons for outpatient pharmacy services satisfaction for insured and noninsured clients under CBHI scheme in Ethiopia. In particular, no similar study has been conducted on patients’ satisfaction with pharmacy services in Gamo zone among users and nonusers of the CBHI program. Therefore, this study is aimed to compare the level of institutional based clients’ satisfaction, and to assess associated factors for satisfaction of insured and noninsured clients under CBHI scheme in Gamo zone public health facilities, South Ethiopia. So, this study fills the gaps that ultimately lead to the desired quality of pharmacy services for patients and raises the degree of patient satisfaction by offering evidence-based information to improve the delivery of health care services in public health facilities.

## Methods

### Study area and period

A facility based cross sectional study was conducted in the selected public health facilities at Gamo zone, which is geographically located in southern region of Ethiopia. The study was conducted in public health facilities at Gamo zone from June 1 to 30, 2023. There are fifty-nine health centers, five primary level hospitals, and one general level hospital in the Gamo zone. Various pharmacy services are offered by the general hospital, such as inpatient, outpatient, emergency, obstetrics and gynecology, mother and child healthcare, and chronic care pharmacy. Pharmacy services are offered by primary level hospitals as inpatient, outpatient, and emergency and chronic care options. Only patients can receive outpatient pharmacy services from health centers.

### Study design

A concurrent mixed method was applied for quantitative survey and qualitative study. The quantitative method was used to compare the level of satisfaction between insured and noninsured clients under CBHI scheme, whereas qualitative method was used to explore the experiences with a pharmacy services offered at outpatient department pharmacy in public health facilities.

### Populations

#### Source population

All patients who visited the OPD (outpatient department) pharmacy of the study health institutions were included in the source population for this study.

### Study population

Adult CBHI members and non-members who received pharmacy services from the OPD pharmacy in the selected public health facilities during the study period were included as the study population.

### Eligibility criteria

#### Inclusion criteria

The survey comprised both insured and uninsured patients who visited the public health facilities’ OPD pharmacies during the study period. Patients who were willing to participate in the study and the years *≥* 18 were also enrolled, but for those below 18 years the caregiver or guardians were included.

#### Exclusion criteria

Patients who were treated in other pharmacy departments (out of OPD), patients with hearing, and mental impairments, and unwillingness to give consent were excluded from the study.

### Study variables

There are two variables; dependent (outcome) variable and independent (explanatory) variables.

### Dependent variable

- Client satisfaction with pharmacy service.

### Independent variables

#### Independent variables for insured clients

- **Socio-demographic characteristics**: Age, gender, marital status, educational level, occupation, religion, place of residence, & monthly income.
- **CBHI package related factors**: CBHI package availability, knowledge on CBHI package, & premium affordability of the scheme.
- **Service related factors**: Pharmacy staff communication, service equality, pharmacist dignity and respect & waiting time.
- **Products related factors**: Availability of supplies & quality of perceived products.

#### Independent variables for noninsured clients

- **Socio-demographic characteristics**: Age, gender, marital status, educational level, occupation, religion, place of residence, & monthly income.
- **Service related factors**: Pharmacy staff communication, service equality, pharmacist dignity and respect & waiting time.
- **Products related factors**: Availability of supplies, quality of perceived products & cost of pharmaceuticals.

### Sample size determination and sampling procedures

#### Sample size determination for health facilities (HFs)

WHO recommended for assessment of health facilities by considering the available funds and human resources, selecting 10-50% facilities to have representative sample [16].

Among the total of 34 health facilities in the selected districts, 30% of health facilities in study districts were selected based on the suggestion. A total of 10 health facilities were selected by simple random sampling using lottery method.

### Sampling procedures for HFs

To insure the representativeness of the sample, the health facilities under each districts and town administrations were stratified, then the sample of the facilities were selected randomly, and the redundant selection of health facilities from one district was managed by identifying the pool for selection (Table 1).

**Table 1:** Districts, town administrations and public health facilities selected to compare the levels of client’s satisfaction with pharmacy services among users and nonusers of community based health insurance scheme at Gamo zone, 2023

S/*N*	Name of woredas/town administrations	Selected health facilities
1	Arbaminch Zuria Woreda	Lante Health Center
2	Bonke Woreda	Gezeso Health Center
3	Boreda Woreda	Zefine Health Center
4	Chencha Zuria Woreda	Dorze Health Center
5	Dita Woreda	Andro & Zadha Health Center
6	Gerrese Woreda	Bulla Health Center
7	Mirab Abaya Woreda	Birbir Health Center
8	Arbaminch Town Administration	Arbaminch General Hospital
9	Chencha Town Administration	Chencha Primary Hospital

#### Sample size determination for quantitative study participants

The sample size for this study was calculated manually by using double population proportion formula, which was assumed to be 80% power of study at 95% of confidence interval (CI). The formula used for the calculation is [17]:

$$n\hspace{0.17em}=\hspace{0.17em}\frac{p1 \left(1-p1\right)+p2 \left(1-p2\right)*C}{\left(p1-p2\right)2}$$

C = Standard value for the corresponding level of α and β selected for the study.

Based on the following values, the sample size (n) is determined as follow:

P1: is proportion of satisfaction in the CBHI member clients and was taken as 63.4% from the study done at West Arsi Zone, Ethiopia on client satisfaction on community based health insurance scheme and associated factors [16];

P2: is proportion of satisfaction in non CBHI member clients and was taken as 50.9% from a study conducted in Debre Tabor Northwest, Ethiopia on Patients’ satisfaction with outpatient pharmacy services and associated factors [18];

α: the level of statistical significant = 0.05; β: type II error = 0.2; and none response rate at 10%.

C = 7.9, when the power of study is 80% and with confidence interval (CI) of 95%.

Therefore, the final calculated sample size is:

$$n\hspace{0.17em}=\frac{p1 \left(1-p1\right)+p2 \left(1-p2\right)*C}{\left(p1-p2\right)2}\hspace{0.17em}$$

$$\begin {array} {l} =\hspace{0.17em}\frac{0.634*\left(0.366\right)\hspace{0.17em}+\hspace{0.17em}0.509*\left(0.491\right) *7.9}{(0.634\hspace{0.17em}-\hspace{0.17em}0.509)2}=\hspace{0.17em}244\hspace{0.17em}+\hspace{0.17em}24\\=\hspace{0.17em}268 \left(Sample\, size\, for\, each\, group\right)\end{array}$$

#### Sampling procedures for quantitative study participants

Data was gathered from both insured and uninsured patients who visited the OPD pharmacy during the study period by using a simple random sampling technique. A ratio of insured to non-insured was 1:1.

Study participants were proportionally allocated to each selected health facilities to the both study groups based on the previous month patient flow.

By using proportional probability to size (PPS); = Nf × Ni/N, where,

Nf = final sample size,

Ni = one month report of prior to study in each health facilities, &.

N = total of one month report from each health facilities.

#### Sample size determination for qualitative study participants

Key informant interviews were undertaken to gather qualitative information on patient satisfactions with pharmacy services among community based health insurance scheme users and nonusers. There were different key informants on the defined level of stake. Among this, 16 key informants were included.

This study included carefully chosen key informants, including (insured clients, non-insured clients, pharmacists, the pharmacy head, and CBHI program managers). The sample size for the qualitative study was determined by idea saturation.

#### Sampling procedures for qualitative study participants

Purposive sampling technique was used to select clients from both insured and noninsured group and to select key informants (pharmacists, CBHI program manager, and pharmacy head) in order to explore the experience with a pharmacy services offered at OPD pharmacy for insured & noninsured groups of clients in public health facilities.

### Data collection instrument

#### For quantitative data

The data collection tools for this study were a structured and semi structured interview questionnaire. The questionnaire was prepared after an intensive review of related literature on the topic [7, 13, 14].

The CBHI scheme related part was adopted and developed from the Federal Democratic Republic of Ethiopia, Ethiopian Health Insurance Agency Evaluation of CBHI pilot scheme in Ethiopia and WHO CBHI scheme guideline [15, 16].

There three items of questions were used in five point Likert scale to measure the level of satisfaction for the both groups of clients, such as; study participants’ opinions towards the pharmacy setting, medication availability, and cost, study participants’ satisfaction towards the dispensers approach or communication & study participants’ satisfaction with the pharmaceutical services instructions. The level of client satisfaction was rated out of five. The patients were asked to rate their level of satisfaction on a five-point Likert scale in each of the satisfaction questions (1: very dissatisfied, 2: dissatisfied, 3: neutral, 4: satisfied, and 5: very satisfied).

#### For qualitative data

Semi-structured & interview administered questionnaires was adopted and developed from different literatures [15–17].To gather qualitative data for the study, an open-ended data tool with probes was used.

### Data collection procedure

Patient exit interview (survey) was used for quantitative study, while in-depth interview was applied for the qualitative study.

#### For quantitative study

The data collectors were pharmacy technicians working in the neighboring health facilities and they were trained on the data collection process. A two-day long training was given for the collectors on principles of data collection, components of instruments and ethical principles.

Based on the results of a pretest, tools used in the study was modified by in lining the data tools with specific objectives of the study, and the total time adjustment for the data tool was done, then a patient exit interview survey was used to collect data. The total time to collect the data was 30 min.

Interviewees whose age is 18 years old and above were interviewed, while caretakers or guardians of those who are below 18 years were interviewed.

For supervisory tasks, senior pharmacists were hired. Every day, the supervisors observed the data collection process.

#### For qualitative study

The clients were interviewed through in-depth interview after getting pharmacy services at outpatient department pharmacy. Field note for each questions and responses was taken in local language (Gaamotho) and an audio recorder was used. Interviewee guide with probes was used to clarify and expand on the key informant’s response. The place of interview was at the office of respective key informants, and it was conducted by the principal investigator.

### Data processing, analysis and presentation

#### For quantitative study

After being checked for accuracy and consistency, the data was coded, imported into Epi Data 3.1, and exported to the statistical program SPSS version 23 for analysis. Descriptive statistics (including mean scores, frequencies and percentages) were employed to summarize the socio-demographic characteristics of the patients and facility-related factors.

The collected data’s were scored using a five-level Likert scale of categories, then patient satisfaction was assessed by using 22 items of satisfaction measurement. For each group of clients, a mean score was calculated by summing individual perceived scores for each item of the questions under the Likert scale and dividing the result by five.

The five scales were combined into a two-scale structure for descriptive interpretation by calculating the cut point from the mean value by using demarcation threshold formula: {Highest total score – Lowest total score/2} + Total lowest score [16]. Overall customer satisfaction was categorized as “satisfied” for both groups of members if the score was above a predetermined cut point and “dissatisfied” if it was below it. The cut point value of satisfaction level with the pharmacy service was found to be 3.7 for insured & 3.2 for noninsured clients.

The adjusted odds ratio (AOR) was used to measure the association between independent variables and patient satisfaction toward outpatient pharmacy services at the P values < 0.05 and 95% confidence interval (CI).

Binary logistic regression analysis was used to determine associations between the different independent variables and the key outcome variables. Finally the findings were presented using tables, charts, figures, graphs and texts.

#### For qualitative study

The field notes and audio records were transcribed and translated to English for.

further analysis. The data was coded by categorizing the collected non numerical data in to groups and assigning the numerical codes to those groups.

Following the translation and transcribing of the data into multiple codes, the qualitative data was analyzed manually by using a thematic analysis technique. Every code was divided into four categories, and each category was then divided into themes by collating together the results of the coding process, generating themes that tie together the identified codes in to groups according to their meaning or subject matter.

### Data quality management

#### For quantitative study

After being created in English, the questionnaire was translated into the local language by those proficient in the language, and its consistency was verified. Before the real data collection period, the 5% of sample was pre-tested for the both groups of clients in the similar setting of the randomly selected health facilities to assess clarity, consistency, understandability of the data tool. Each scale’s reliability was assessed separately. To evaluate the reliability of components, Cronbach alpha coefficients were computed. The Cronbach alpha coefficient values indicate that it was within the acceptable alpha value range (> 0.7). Then, the necessary comments and feedbacks were incorporated into the final tool to improve its quality.

#### For qualitative study

The trustworthiness parameters (credibility, transferability, conformability, and dependability) were ensured through participant validation, peer debriefing, informant feedback, and prolonged time spent with the key informants.

Appointment times were made up to ensure continued communication between the investigator and the study’s key informants. The specifics of the situation were noted (note-taking with probes & audio recording were employed). Through the employment of various key informant categories at various sites, triangulation was made.

## Results

This section includes the results of both the survey & qualitative study. A total of 522 respondents were included in the quantitative study, with a response rate of 97.3%.

### Quantitative finding

#### Socio-demographic characteristics of the study participants

Among the total of study participants, 264 (49.2%) were insured and 258 (48.1%) were non-insured under the CBHI scheme.

From the study groups, the most of insured clients 109 (41.3%) were under the age category of 26–35, similarly 84 (32.6%) of noninsured clients were under this age category.

Based on the educational level, the majority study participants for insured groups 129 (48.9%) were illiterate, while 165 (64%) of noninsured were had certificate & above. Those from insured groups, the highest number of clients 90 (34.1%) were farmer, but conversely 129 (50%) from noninsured groups were government employee.

In terms of the marital status, the copious of the insured study participants 190 (72%) were married, in the same way the high amount of 162 (62.8%) noninsured study participants were married (Table 2).

**Table 2:** Socio-demographic characteristics of the study participants at visiting outpatient pharmacy department with pharmacy services in public health facilities at Gamo zone southern, Ethiopia; 2023

Variables	Response category	Insured (*n* = 264)		Noninsured (*n* = 258)	
		Frequency	%	Frequency	%
Age	18–25	68	25.8	60	23.3
	26–35	109	41.3	84	32.6
	36–50	66	25.0	81	31.4
	> 50	21	8.0	33	12.8
Gender	Male	138	52.3	172	66.7
	Female	126	47.7	86	33.3
Educational status	No formal education	129	48.9	6	2.3
	Primary education	90	34.1	31	12.0
	Secondary education	34	12.9	56	21.7
	Certificate and above	11	4.2	165	64.0
Religion	Protestant	119	45.1	127	49.2
	Orthodox	100	37.9	91	35.3
	Muslim	29	11.0	22	8.5
	Catholic	10	3.8	13	5.0
	Other	6	2.3	5	1.9
Marital status	Single	46	17.4	75	29.1
	Married	190	72.0	162	62.8
	Divorced/Separated	9	3.4	3	1.2
	Widowed	19	7.2	18	7.0
Occupation	Farmer	90	34.1	9	3.5
	No job	89	33.7	25	9.7
	Merchant	43	16.3	20	7.8
	Other	42	15.9	204	79.1
Residence	Urban	155	58.7	190	73.6
	Rural	109	41.3	68	26.4
Monthly income	< 1000	119	45.1	58	22.5
	1001–5000	125	47.3	73	28.3
	5001–10,000	16	6.1	115	44.6
	> 10,001	4	1.5	12	4.7

#### Study participants’ opinions towards the pharmacy setting, medication availability, and cost

From the study participants, many of 246 (93.2%) the insured study participants were satisfied with pharmacy location convenience, likewise a large number 190 (73.6%) of noninsured participants were also satisfied with pharmacy location convenience. Similarly, both groups of clients were highly dissatisfied with the pharmaceuticals unavailability in the dispensary units, which was 85 (32.2%) for insured & 130 (50.4%) for uninsured groups. Nearly amount, 184 (69.7%) of insured and 163 (63.2%) of noninsured clients were satisfied with enough waiting seat in the waiting area (Table 3).

**Table 3:** Study participants’ opinions towards the pharmacy setting, medication availability at Gamo zone public health facilities, 2023

Variables for level of satisfaction	Insured (*n* = 264)		Noninsured (*n* = 258)	
	Satisfied *n* (%)	Dissatisfied *n* (%)	Satisfied *n* (%)	Dissatisfied *n* (%)
The pharmacy location convenience	246 (93.2)	18 (6.8)	190 (73.6)	68 (26.4)
Enough waiting seat in the waiting area	184 (69.7)	80 (30.3)	163 (63.2)	95 (36.8)
Waiting area is comfortable & convenient	191 (72.3)	73 (27.7)	165 (64.0)	93 (36.0)
Pharmacy service area is clean	243 (92.0)	21 (8.0)	190 (73.6)	68 (26.4)
Counseling area is comfortable and convenience	241 (91.3)	23 (8.7)	189 (73.3)	69 (26.7)
Pharmaceuticals are available	179 (67.8)	85 (32.2)	128 (49.6)	130 (50.4)
Enough number of staffs to provide pharmacy service	237 (89.8)	27 (10.2)	182 (70.5)	76 (29.5)

#### Study participants’ satisfaction towards the dispensers approach or communication

Of the respondents, a large number of 248 (96.2%) the insured groups were satisfied with dispenser’s availability in dispensary unit, in the same way the majority 208 (80.6%) of noninsured groups were satisfied with dispenser’s availability in dispensary unit. From both groups of study participants, a great number 114 (43.2%) of insured & 123 (47.7%) of noninsured clients were highly dissatisfied by a chance given by the dispensers to ask a question on pharmaceuticals and any ambiguity (Table 4).

**Table 4:** Study participants’ satisfaction towards the dispensers approach or communication at Gamo zone public health facilities, 2023

Variables for level of satisfaction	Insured (*n* = 264)		Noninsured (*n* = 258)	
	Satisfied *n* (%)	Dissatisfied *n* (%)	Satisfied *n* (%)	Dissatisfied *n* (%)
Dispensers show politeness and interest to serve clients	239 (90.5)	25 (9.5)	183 (70.9)	75 (29.1)
Dispenser provide service equally for all clients without any favor	197 (74.6)	67 (25.4)	189 (73.3)	69 (26.7)
Dispensers treat clients with dignity and respect	244 (92.4)	20 (7.6)	200 (77.5)	58 (22.5)
Dispensers availability in dispensary unit	254 (96.2)	10 (3.8)	208 (80.6)	50 (19.4)
Dispensers clarity of the voice and tone	248 (93.9)	16 (6.1)	204 (79.1)	54 (20.9)
Dispenser fairness of providing service waiting time in the pharmacy	244 (92.4)	20 (7.6)	203 (78.7)	55 (21.3)
Dispenser gave me a chance to ask a question on my pharmaceuticals and any ambiguity	150 (56.8)	114 (43.2)	135 (52.3)	123 (47.7)

#### Study participants’ satisfaction with the pharmacy services instructions

From the study groups, the masses 248 (93.9%) of insured were highly satisfied with the language used by dispensers on administration of pharmaceuticals, but conversely 208 (80.6%) noninsured were highly satisfied with the adequate explanations provided by the dispensers on how to use pharmaceuticals According to the important drug and health related history questions asked by the dispensers on pharmaceuticals, more than the halves 174 (65.9%) of insured and 145 (56.2%) of noninsured study participants were dissatisfied in this study (Table 5).

**Table 5:** Study participants’ satisfaction with the pharmacy services instructions at Gamo zone public health facilities, 2023

Variables for level of satisfaction	Insured (*n* = 264)		Noninsured (*n* = 258)	
	Satisfied *n* (%)	Dissatisfied *n* (%)	Satisfied *n* (%)	Dissatisfied *n* (%)
Dispenser asked me important drug and health related history	90 (34.1)	174 (65.9)	89 (34.5)	145 (56.2)
Dispenser provides adequate explanation on how to use my pharmaceuticals	244 (92.4)	20 (7.6)	208 (80.6)	60 (23.3)
Dispenser mentions information about drug-drug and drug-food interaction	228 (86.4)	36 (13.6)	192 (74.4)	66 (25.6)
Dispenser provide adequate information on proper storage of pharmaceuticals	230 (87.1)	34 (12.9)	172 (66.7)	86 (33.3)
Dispenser told me about pharmaceuticals precautions and side effects	217 (82.2)	47 (17.8)	187 (72.5)	71 (27.5)
Dispenser’s labeling to the pharmaceuticals is readable and understandable	232 (87.9)	32 (12.1)	180 (69.8)	78 (30.2)
Language used by dispenser on administration of pharmaceuticals is easy and understandable	248 (93.9)	16 (6.1)	198 (76.7)	50 (19.4)
Dispenser tried to make sure, if I understand how to take my pharmaceutical products	155 (58.7)	109 (41.3)	113 (43.8)	169 (65.5)

#### Perceived level of satisfactions and dissatisfactions between insured & noninsured study participants

**Fig. 1:**
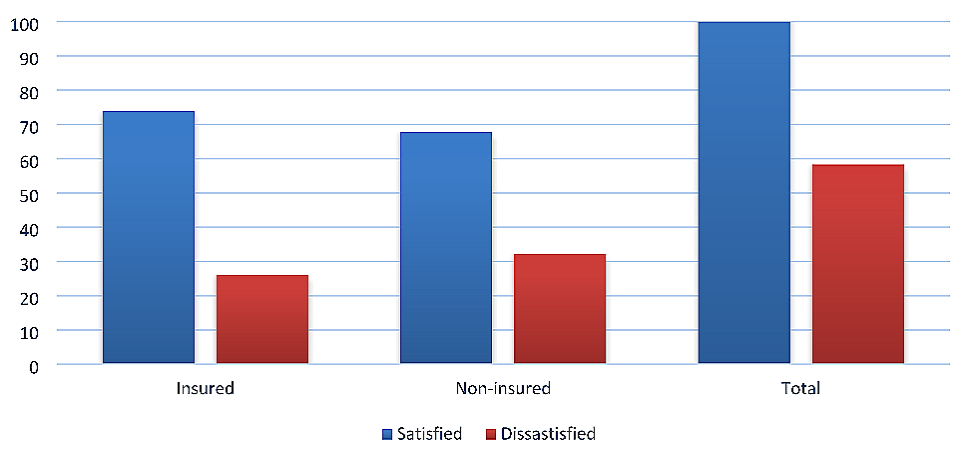
Perceived level of satisfactions & dissatisfaction for insured & noninsured groups with pharmacy services at Gamo zone public health facilities, 2023

From the total of study participants, 195 (73.9%) of insured & 175 (67.8%) of noninsured clients were satisfied, while 69 (26.1%) of insured & 83 (32.2%) noninsured groups were dissatisfied with pharmacy services offered at public health facilities (Fig. 1).

#### Factors associated with client satisfaction on pharmacy services at public health facilities

In order to identify the variables influencing overall patient satisfaction among patients covered by the CBHI scheme as well as those who are not, bivariate and multivariate logistic regression analyses were conducted. The study variables with a p-value < 0.25 in the bivariate model were included in the final multivariate model.

At the bivariate logistic regression model, the socio-demographic variables; age (95% CI = 1.641–15.958, p (0.005)), and gender (95% CI = 0.897–2.731, p (0.114)), availability of products (95% CI = 1.006–9.295,p ( 0.049)) & waiting times (95% CI = 0.146–1.392, p (0.166)) for both groups of clients and the CBHI package availability (95% CI = (0.173-0.625, p (0.001)) and premium affordability (95% CI = (1.945–8.716, p (0.00)) for the insured groups of clients were a significant factors associated with the client satisfactions at *p* < 0.25 & 95% CI, and chosen as candidate variables for multiple variable analysis (Table 6).

**Table 6:** Bivariate & Multivariate logistic regression results on pharmacy services of insured groups of patients in Gamo zone, Southern Ethiopia, 2023

Variables		Satisfaction Category		COR (95% CI)	*P*- value	AOR (95% CI)	*P*- value
		Satisfied	Dissatisfied				
Gender	Male	80	72	1^R^		1^R^	
	Female	62	50	1.565(0.897–2.731)	0.114	3.34 (2.00–12.36)	0.010*
Age	18–25	35	22	1^R^		1^R^	
	26–35	54	33	5.117(1.641–15.958)	0.005	5.796(1.511–22.227)	0.396
	36–50	46	28	7.789(2.591–23.418)	0.000	7.625(2.092–27.789)	0.811
	> 50	29	17	1.943(1.141–3.308)	0.01	4.052(1.009–13.312)	0.322
Availability of products	Good	152	60	1R		1^R^	
	I don’t know	20	14	3.058(1.006–9.295)	0.049	1.712(0.414-7.085).	0.432
	Not good	16	2	0.439(0.248-0.776)	0.005	2.723(0.542–6.966)	0.210
Waiting time	*≤* 15 min	180	30	1^R^		1^R^	
	>15 min	32	22	0.451(0.146-1.392)	0.166	1.65(0.057–0.766)	0.0027*
CBHI package availability	Good	120	82	1^R^		1^R^	
	Not good	38	24	0.329(0.173-0.625)	0.001	0.680(0.317–1.459)	0.310
Premium affordability	Affordable	100	55	1^R^		1^R^	
	Not affordable	68	41	4.117(1.945–8.716)	0.00	1.715(0.605–4.860)	0.000*

COR - crude odds ratio, AOR- adjusted odds ratio, 1R reference category * p- value < 0.05

The socio-demographic factors and other significant variables from the bivariate logistic regression model did not correlate with the level of client satisfaction at (p-value < 0.05) in the multivariate logistic regression model. However, gender of insured (95% CI = 2.00–12.36, (p 0.01)), & noninsured (95% CI = 0.658–2.881, (p 0.02)), waiting time of insured (95% CI = 0.057–0.766, (p 0.0027)), & noninsured (95% CI = 0.084–0.925, (p 0. 0021)) and premium affordability (95% CI = 0.0605–4.860, (p 0.00)), were significantly associated factors with the outcome variables at *p* < 0.05 & 95% CI (Table 7).

**Table 7:** Bivariate & Multivariate logistic regression results on pharmacy services of noninsured groups of patients in Gamo zone, Southern Ethiopia, 2023

Variables		Satisfaction Category		COR (95% CI)	*P*- value	AOR (95% CI)	*P*- value
		Satisfied	Dissatisfied				
Gender	Male	102	70	1^R^		1^R^	
	Female	58	28	1.565(0.897–2.731)	0.114	1.377(0.658–2.881)	0.020*
Age	18–25	34	26	1^R^		1^R^	
	26–35	49	35	5.117(1.641–15.958)	0.005	5.796(1.511–22.227)	0.396
	36–50	42	39	7.789(2.591–23.418)	0.000	7.625(2.092–27.789)	0.811
	> 50	20	13	1.943(1.141–3.308)	0.01	4.052(1.009–13.312)	0.322
Availability of products	Good	120	89	1R		1^R^	
	I don’t know	36	12	3.058(1.006–9.295)	0.049	1.712(0.414-7.085).	0.432
	Not good	1	0	0.439(0.248-0.776)	0.005	2.723(0.542–6.966)	0.210
Waiting time	*≤* 15 min	112	76	1^R^		1^R^	
	>15 min	55	15	0.451(0.146-1.392)	0.166	2.97(0.084–0.925)	0.0021*

COR - crude odds ratio, AOR- adjusted odds ratio, 1^R^ reference category * p- value < 0.05

#### Patient experiences of insured & noninsured clients with pharmacy services in public health facilities

Regarding to the familiarity with institution, more than the three quarters of 229 (86.7%) insured and noninsured 208 (80.6%) groups were chronic users for the health facilities. Concerning their number of visit to the facilities of the study participants, the highest number 108 (40.9%) of insured clients were visited the public health facilities for *≥* four time, while the greatest number 87 (33.7%) of insured were visited for the first time. More than the half of insured 210 (79%) and noninsured groups 188 (72.9%) were getting their pharmacy services within less or equal of 15 min at outpatient pharmacy department (Table 8).

**Table 8:** Study participant’s experiences with pharmacy services at Gamo zone of public health facilities in Southern Ethiopia, 2023

Variables	Response Category	Group Category	
		Insured (*n* = 264) n (%)	Noninsured(*n* = 258) n (%)
Service sought for	Primary healthcare	189 (71.6)	217 (84.1)
	Dental care	44 (16.7)	12 (4.7)
	Family planning & consultation	19 (7.2)	15 (5.8)
	Optical care	8 (3.0)	11 (4.3)
	Others	4 (1.5)	3 (1.2)
Familiarity with institution	First visit	35 (13.3)	50 (19.4)
	Chronic user	229 (86.7)	208 (80.6)
Number of visit in this year	First time	47 (17.8)	87 (33.7)
	Second time	60 (22.7)	63 (24.4)
	Third time	49 (18.6)	49 (19.0)
	*≥* Four time	108 (40.9)	59 (22.9)
Availability of pharmaceuticals	Good	212 (80.3)	209 (81.0)
	I don’t know	34 (12.9)	48 (18.6)
	Not good	18 (6.8)	1 (0.4)
Waiting time	*≤* 15 min	210 (79)	188 (72.9)
	> 15 min	54 [20]	70 (27.1)

#### Views of clients regarding the premium package and benefits of the CBHI scheme implementation (for CBHI members)

Regarding to CBHI package accessibility, out of 264 study participants, 202 (76.5%) of.

them were have had a good accessibility. Concerning the CBHI package benefit, 254 (96.2%) of the clients were getting a very adequate benefit. More than half of the study participants (155, 58.7%) were perceived that the premium paid to the scheme is affordable. Two hundred sixty three (99.6%) of the clients had intend to extend membership status (Table 9).

**Table 9:** Views of clients regarding the premium package and benefits of the CBHI scheme implementation at Gamo zone, Southern Ethiopia 2023

Variables	Response Category	Frequency	Percentage
CBHI package accessibility	Good	202	76.5
	Not good	62	23.5
CBHI package benefit	Very adequate	254	96.2
	Somewhat adequate	9	3.4
	Inadequate	1	0.4
Premium affordability	Affordable	155	58.7
	Not affordable	109	41.3
Plan to renew membership	Yes	263	99.6
	No	1	0.4
Preference of health facilities for next use	Yes	264	100
	No	0	0

### Qualitative finding

In this section of the study, purposively selected key informants were included for the data gathering. There were different key informants on the defined level of stake (insured and noninsured clients, pharmacists, pharmacy heads, and program managers for the CBHI scheme).

#### Study participant’s experiences toward CBHI program & pharmacy services

Patient experience means varied interactions that the patients have with the healthcare systems, so the clients experience with the CBHI package in public health facilities should be used to evaluate the quality of services offered & the level of satisfactions.

“……*With the respect to the experience with CBHI services, being the member of CBHI scheme give us a chance to get good service by increasing the familiarity with the institutions as well as with the physicians. That results in good levels of satisfactions on the services provided.*

[Insured Patient, Female (F), 38 years old]

……According to the experience with the pharmacy services, the way of approaches & communication of the pharmacists in public health facilities is very good and that yields in increasing the familiarity with the institutions.” In terms of friendly relationships with pharmacists, the pharmacy services offered to us are in nice manner and getting them within the short minutes of time intervals.

[Noninsured Patient, Male (M), 35 years old]

The physicians toward the experiences of CBHI package services indicated that, most of insured clients come to the health facilities with un renewed ID cards, they are familiar with the institution and the services

………With the regard to the activities to be added to serve the package users in an upstanding manner, increasing the number of employee and dispensary units should be taken as a good option. The incompatible number of pharmacists and the CBHI user’s upshot in workload for the pharmacists & negative effects on the quality of the services offered.

[25 years old Male BSC Pharmacist]

*“……The availability and applying this CBHI scheme in our country was very important for the low level income patients. With respect to physicians and government, obviously it increased the workload and result in shortage of budget due to current inflation of pharmaceutical products, respectively.”*

[35 years old Male Pharmacy Head]

*“……According to the experiences toward CBHI package services; the CBHI scheme was applied to the south region in 2003 EC and implemented in 2008 EC at Gamo zone in three woredas, and now currently applicable to all woredas throughout the zone.”*

[40 years old Male CBHI Program Manager]

### Benefits of implementing CBHI program

The introduction of the CBHI scheme has reduced catastrophic out of pocket (OOP) expenditure, increased healthcare utilization, and the quality of the services through retaining mobilized resources at the public health facilities.

…….Based on the advantages, it’s very important especially for the persons who had low income level, this is due to we are getting many healthcare services with optimum premium payment and with respect to disadvantages, the users said that some of the pharmacists give priority to the noninsured clients since they are getting the service by paying at the point of consumption.

[Insured Patient, Female (F), 30 years (yrs.) old]

……. With the regard to the benefits of CBHI scheme, it’s is very crucial for clients that they can’t offer from the out of pocket to get healthcare services and also too cost effective for the users. The demerits with respect to physicians, it increases the workload there by resulting in arising questions like: payment of overtime/OT and employment of new employee.

[24 years old Female Pharmacy Head]

……….The CBHI package is useful for the users due to its cost effectiveness with symmetric annual payment and they get many healthcare services including; inpatient and outpatient services, getting the services by the refer from the other areas, making the physicians free from the stress ( especially for the critical patients) and increased satisfaction for users. The disadvantages are the lack of transport for the CBHI program staffs, lack of on time payment from woredas & insufficient wage/additional payment for the staffs with the respect to the workload.

[30 years old Male CBHI Program Manager]

### Challenges with CBHI program

The enrollment in CBHI schemes is widely challenged by the low perceived quality of care in public health facilities mainly due to shortages of medicines, medical supplies, reagents and diagnostic services, as well as poor referral systems.

…….With the regard to the challenges, the most common challenges are; unavailability of pharmaceuticals, increased waiting time, giving priority to noninsured clients since they were getting services by paying at SDP(service delivery points) and the every yearly increment of premium payment and inaccessibility of the transport services when it comes to refer from the other zones/woredas.

[Insured Patient, Male (M), 28 years old]

*“…….The most common challenges associated with CBHI scheme services include; increment of workload (serving clients, registration & documentation of files) and overcrowdings of clients under pharmacy units and solutions mentioned to overcome the above challenges are increasing the human power, preparing the separated dispensary units for the insured clients and making the systems computerized to provide automated services.”*

[30 years old Male BSC Pharmacist]

…….The common challenges with the CBHI package services includes; the increment of the cost of pharmaceutical products & the shortage of budget due to only 25% of premium payment to the health facilities from the zone health department. The unsymmetrical rule and regulations throughout the regions and zones are also another provocation with the CBHI package services.

[28 years old Male Pharmacy Head]

…….The CBHI package doesn’t include maternal & childcare services, so the clients with low income will render in to another problems (morbidity & mortality), there is lack of supplies due to insufficient budget, lack of awareness by the community on the CBHI scheme, lack of obligations by the physicians in serving clients equally, late payment by the woredas to the zone health department, the income (premium payment) and expense (services) are not proportionate. This results in difficulty for the CBHI program offices and the government to serve the package users in good approach, as well as in nice quality.

[26 years old Male CBHI Program Manager]

#### Solution options for the challenges related with CBHI package

Providing on time solutions for the given problems in public health facilities is very important in controlling and managing diseases, illness and injuries by using a variety of services like; promotion, prevention and intervention in a variety of settings.

The major solutions for the above challenges mentioned by the study participants, includes;

……. The concerning bodies should avail the essential supplies and the transport services, the increment of physicians & dispensary units to decrease waiting time in the pharmacy departments and further discussion with the members before increasing the premium payment.

[Insured Patient, Female (M), 42 years (yrs.) old]

….Solutions indicated to overcome the above challenges are increasing the human power, preparing the separated dispensary units for the insured clients and making the systems computerized to provide automated services.

[22 years old Female BSC Pharmacist]

….The mechanisms mentioned to reduce the above challenges should be including very basic services in to the package, availing the essential products to the public health facilities and increasing the number of healthcare providers and dispensary units to reduce the waiting time, controlling the market situation and correcting the premium payment mechanisms by discussing with the concerning bodies.

[26 years old Male Pharmacy Head]

……The solutions for the common challenges, include separating the dispensary unit for users, creating awareness to the communities by the committee members throughout using media and banners, increasing budget and collecting the premium payment from the members on time. In order to avail the supplies, discussing with Red Cross Arbaminch branch, preparing the community pharmacy with collaboration of Arbaminch University & Arbaminch College of Health Science and dealing with Arbaminch EPSS branch to get supplies with an optimum costs.

[42 years old Male CBHI Program Manager]

## Discussion

This study indicates that there is a significant variation in the perceived level of satisfaction between patients with insured and not insured under the CBHI scheme.

### Perceived levels of satisfactions between insured & noninsured groups of clients with pharmacy services

Among the surveyed study participants, the overall levels of satisfactions of insured clients with the pharmacy services they received at the public health facilities was 73.9%, and this result is higher than reports from other study conducted in West Arsi health centers which showed 63.4% [16], and lower than the study conducted in Boru Meda Hospital, Northeast, Ethiopia which was 80% of overall client satisfaction [15], while the overall level satisfaction of the noninsured clients with pharmacy service was 67.8% and this finding is also higher than the studies done in Dubti General Hospital, Afar, North East Ethiopia which showed 40.5% [18], and lower than the study done in North East Ethiopia which was 75.7% of level of satisfaction with offered services [19].

The deviation in the levels of satisfaction may be due to the clients familiarity with the most of health facilities, the pharmacy staffs dignity & respect during service time for the increased levels of satisfactions, and the unavailability of pharmaceuticals, increased waiting time in outpatient department pharmacy, and the every yearly increment of premium payment for the low levels of satisfactions with the pharmacy services offered at the public health facilities as supported by qualitative investigation. Nonetheless, the study’s uninsured participants reported a high degree of satisfaction with the pharmacy’s instructions about the crucial questions they were asked by the dispensers regarding their medical and drug-related informations. The possibility that non-insured clients were given preference by dispensers since they were paying for services at service delivery points (SDPs) could be the cause of the low level of satisfaction with drug counseling.Hence, in order to provide pharmacy services to all client groups equally, the Ethiopian Federal Ministry of Health (FMOH) in coordination with the South Ethiopia Regional Health Bureau should conduct awareness-raising workshops and offer supportive supervision.

The study findings indicate that slightly more than three-fourths of patients (70.8%) were satisfied with the pharmacy services provided by outpatient department pharmacy. However, it was also show that a significant number (73.9%) of insured patients were satisfied compared to 67.8% of uninsured clients. This result is almost in line with a survey done in North Ethiopia, where 79.4% of insured and 75.7% of non-insured clients were satisfied with the pharmacy services they received [20]. This difference is may be due to the time gap between study times and the study designs conducted.

The study’s reported magnitude of satisfaction score suggests a low proportion of insured patients when compared to other studies, with 82% India, 88% Ghana, 80% Boru Meda, and 79.4% North Ethiopia, while 73% India, 86% Ghana, and 75.7% North Ethiopia had low proportions of noninsured clients when compared to other studies done in the global community and in Ethiopia [16–18, 20]. However, the result of this study shows higher overall patient satisfaction proportion when compared with another study conducted in Brazil where 58.4% of noninsured and in West Arsi 63.4% of insured [11, 21]. This discrepancy might be due to the reasons explained by the study participants in qualitative investigations as the pharmacist’s degree of service quality, the amount of time spent for advising patients, the language and communication skills, the attitude of the pharmacist, and the amount of waiting time in the outpatient pharmacy indicated as a major reasons for the satisfactions & dissatisfactions variations with the pharmacy services in this study. Therefore, the Ethiopian Federal Ministry of Health (FMOH) in collaboration with regional health bureau should expand CBHI package program to all Zones in Ethiopia to reduce the financial risk related to health. Additionally, the FMOH was also more adept at developing and implementing plans to increase public awareness of insurance and provide a sufficient supply of pharmaceuticals in the public health facilities.

### Factors associated with client’s satisfaction on pharmacy services

The findings of this study showed that, there was statistical significant association between gender, waiting time in outpatient pharmacy department, and the premium payment for community based health insurance scheme users with patient satisfaction on pharmacy services.

In this study, from the socio-demographic factors the gender have been observed to be highly associated with the outcome variable for noninsured, those who were insured had a 3.34 times higher level of satisfaction on pharmacy services than those who didn’t insured under CBHI scheme. This difference may be due to the difference in study group and sample size.

Regarding to the waiting time, the waiting time they spent in outpatient pharmacy is also associated with the client’s satisfaction, those who were noninsured had 2.97 times higher satisfaction level on pharmacy services with the waiting time they spent in outpatient pharmacy department than those who were insured under CBHI package. The significance level of satisfaction difference in two groups might be due to the customer’s relations with pharmacy staffs in public health facilities.

The result of the study revealed that, the premium affordability for the insured groups of patients is significantly associated with the satisfaction on pharmacy services at p value of (0.000), the finding of this study indicate the higher associations between the variables than the study conducted in Boru Meda Hospital, Northeast, Ethiopia, which was P -value < 0.05 [15]. This discrepancy may be due to the different amount of premium payment from one region to another region.

### Patient experiences with pharmacy services in public health facilities

The degree to which a patient’s actual experience and expectations with the pharmacy service align is reflected in their level of satisfaction. The finding of this study revealed that, with regard to the familiarity with health facilities, the 86.7% of insured & 80.6% of noninsured clients were a chronic users for the institutions with pharmacy services, and this results are higher than reports from other studies done in Nigeria teaching hospitals, which showed 56.04% [21], and also higher than the study conducted in Debre Tabor comprehensive specialized hospital, Northwest Ethiopia, which was 59.1% [22].

Of the respondents, 79% of insured and 72.9% of noninsured clients were getting their pharmacy services within less or equal of 15 min at outpatient pharmacy department, this findings are higher than the study conducted in in Debre Tabor comprehensive specialized hospital, Northwest Ethiopia, which was 43.6% [22], and lower than the study done in Eastern Ethiopia public hospitals, showed that 79.36% [23]. This difference of client’s experiences with pharmacy services with the current study may be due to the pharmacists the way of approaches & communications, the familiarity with the institutions & in terms of friendly relationships with pharmacists getting the pharmacy services within the short minutes of time intervals as the reasons explained in qualitative investigation.

## Conclusion

Our finding indicates that patients with insurance perceived high level of satisfaction on pharmacy services than non-insured. However, noninsured study participants received high level of satisfaction on instructions with pharmacy services of important questions asked by the dispensers on drug and health related histories. Some of socio-demographic factors, availability of products & waiting times for both groups of study participants and the CBHI package availability and premium affordability for the insured groups of clients were a significant factors associated with the client satisfactions with pharmacy services.

The clients under CBHI scheme were familiar with pharmacy services in public health facilities than noninsured groups of clients. Nevertheless, the noninsured study participants were get good pharmaceutical products than insured groups of clients in public health facilities.

According to the qualitative finding of the study, the implementation CBHI package have both advantages and disadvantages to the patients, pharmacy staffs, pharmacy heads & CBHI program planers. Therefore, the qualitative study was very important to understand the experiences and challenges related with the CBHI scheme for the clients, pharmacists, pharmacy heads and CBHI program managers.

## Data Availability

Data is available upon request to the corresponding author.

## Declarations

### Abbrievations

AOR: Adjusted Odd Ratio.

CBHI: Community Based Health Insurance.

CI: Confidence Interval.

COR: Crude Odd Ratio.

HC: Health Center.

HF: Health Facility.

SRS: Simple Random Sampling.

OPD: Outpatient Department.

PPS: Proportional Probability to Size.

SDP: Service Delivery Point.

UHC: Universal Health Coverage.

WHO: World Health Organization.
